# L-Arginine supplementation in mice enhances NO production in spleen cells and inhibits *Plasmodium yoelii* transmission in mosquitoes

**DOI:** 10.1186/s13071-015-0940-0

**Published:** 2015-06-14

**Authors:** Li Zheng, Yanyan Pan, Yonghui Feng, Liwang Cui, Yaming Cao

**Affiliations:** Department of Immunology, College of Basic Medical Sciences, China Medical University, No.77 Puhe Road, Shenyang North New Area, Shenyang, Liaoning 110122 P. R. China; Department of Entomology, Pennsylvania State University, University Park, PA USA

**Keywords:** *Plasmodium yoelii*, L-Arginine, Immune regulation, Blood stage, Mosquito

## Abstract

**Background:**

The life cycle of *Plasmodium* is complex, requiring invasion of two different hosts, humans and mosquitoes. In humans, initiation of an effective Th1 response during early infection is critical for the control of parasite multiplication. In mosquitoes, inhibition of the development of sexual-stage parasites interrupts the parasite transmission. In this study, we aim to investigate whether dietary supplementation of L-arginine (L-Arg) in mice affects *Plasmodium yoelii* 17XL (*Py*17XL) transmission in mosquitoes.

**Methods:**

BALB/c mice were orally administered with 1.5 mg/g L-Arg daily for 7 days and infected with *Py*17XL. The mRNA levels of *inducible nitric oxide synthase* (*iNOS*) and *arginase 1* in spleen cells were determined by real-time RT-PCR. The amount of nitric oxide (NO) released by spleen cells in vitro was determined by the Griess method. The effect of L-Arg supplementation on subsequent development of *P. yoelii* gametocytes was evaluated by an in vitro ookinete culture assay and mosquito feeding assay.

**Results:**

Pretreatment of mice with L-Arg significantly increased the transcript level of *iNOS* in spleen cells and the amount of NO synthesized. Dietary L-Arg supplementation also significantly reduced the number of zygotes and ookinetes formed during in vitro culture and the number of oocysts formed on mosquito midguts after blood feeding.

**Conclusions:**

L-Arg enhances host immunity against blood-stage parasites as well as suppressing subsequent parasite development in mosquitoes. L-Arg as an inexpensive and safe supplement may be used as a novel adjunct treatment against malarial infection.

## Background

Malaria is a serious vector-borne disease that accounts for over half a million deaths annually. This infection not only remains widespread throughout the tropics, but also occurs in many temperate regions [1]. The intolerable health burden of malaria and its socio-economic impacts have inspired the development of a Global Malaria Action Plan by the World Health Organization (WHO) [2], which has re-established elimination and eradication on the malaria control agenda.

The life cycle of *Plasmodium* is complex. Usually, people get malaria after being bitten by an infected female *Anopheles* mosquito. The parasites first multiply in the human liver and subsequently infect red blood cells. All symptoms of malaria including fever, anaemia, and neurological pathology, appear during the blood stages of the infection. Therefore, medications are primarily directed against the blood-stage parasites to ameliorate the malaria symptoms, whereas transmission blocking strategies target the sexual stages such as gametocyte, zygote, and ookinete [3, 4].

L-Arginine (L-Arg), an essential amino acid in newborns and infants, is the sole substrate of nitric oxide (NO) synthase required for the production of NO. Hypoargininemia can lead to impaired systemic NO production [5]. Therefore, effects of L-Arg supplementation are mostly likely due to functional enhancement of iNOS activity [6]. Low arginine, low nitric oxide production, and endothelial dysfunction are common in severe malaria [7]. In patients with severe malaria, supplementation with L-Arg has been shown to improve NO bioavailability and reverse endothelial dysfunction [8]. We have recently shown that supplementation of L-Arg promotes the maturation of dendritic cells and improves protective immunity during early-stage *Plasmodium yoelii* 17XL (*Py*17XL) infection [9]. We have also shown that exogenous NO inhibits the development of *P. yoelii* gametocytes into gametes [10]. Direct supplementation of NO is not feasible, as NO is a short-lived free radical and direct NO administration has severe side effects for the host. In contrast, L-Arg is a safe and widely accepted amino acid supplement that has been used in humans for decades [11].

In this study, we studied the effects of L-Arg supplementation on the transcript levels of two arginine-metabolizing enzymes nitric oxide synthase (iNOS) and arginase 1 (ARG1). We then determined the effects of L-Arg supplementation on NO production in splenocyte culture and the development of zygotes and ookinetes in vitro and the formation of oocysts in vivo. Our results confirmed that L-Arg supplementation in mice enhances the expression of *iNOS* and further inhibits the development of sexual stages in mosquitoes.

## Methods

### Mice, parasite, infection, and L-Arg treatment

Female BALB/c mice (6–8 weeks of age) were purchased from the Center of Zoology, Chinese Academy of Sciences. Infections with *Py*17XL were initiated by intraperitoneal injection of 10^6^*Py*17XL parasitized erythrocytes per mouse. Parasitemia was monitored by light microscopy of Giemsa-stained thin blood smears. Mortality was monitored daily. All experiments were performed in compliance with regulations of the animal ethics committee of China Medical University.

L-Arg (Sigma-Aldrich, St. Louis, MO, USA) was dissolved in normal saline prior to use. Mice were administered an oral dose of 1.5 mg/g L-Arg daily for seven consecutive days prior to infection with *Py*17XL. The control group received D-Arg dissolved in the same volume of normal saline at identical time points.

### Splenocyte preparation and culture

Splenocyte culture was prepared as previously described [12]. Splenocytes were adjusted to a final density of 10^7^ cells/mL in RPMI-1640 supplemented with 10 % heat-inactivated fetal calf serum, seeded in 24-well flat-bottom Falcon tissue culture plates (Corning, Tewksbury, MA, USA) in triplicate at 5 × 10^6^/well, and incubated at 37 °C for 48 h in a humidified 5 % CO_2_ incubator. The resulting supernatants were collected and stored at −80 °C for quantification of NO levels.

### RNA extraction and real-time RT-PCR

Total RNA was isolated from spleen cells using TRIzol®. The ratios of absorbance at 260/280 nm ranged from 1.80 to 2.00 for all RNA samples. Total RNA was stored at −70 °C. cDNA was synthesized using the PrimeScript^TM^ RT reagent kit with gDNA Eraser (Takara, Japan). Reverse transcription was performed in a 10 μL reaction mixture containing PrimeScript^TM^ buffer, PrimeScript^TM^ RT enzyme mix, oligo dT primer (50 μM), random hexamers (100 μM), and 500 ng total RNA. PCR was performed using the resulting cDNA as a template and gene-specific primers. Primers used for PCR were as follows: iNOS forward (5′ TCCTCACTGG GACAGCACAGAATG 3′) and reverse (5′ GTGTCATGCAAAATCTCTCCACTGCC 3′); and ARG1 forward (5′ ATGGAAGAGACCTTCAGCTAC 3′) and reverse (5′ GCTGTCTTCCCAAGAGTTGGG 3′). Quantitative PCR was carried out using the SYBR® Premix Ex Taq™ reagent kit (Takara) in an AB7500 instrument (Applied Biosystems, USA). After denaturation at 95 °C for 30 s, 40 cycles of 95 °C for 5 s, followed by 60 °C for 30 s were performed. *β-actin* was used as an internal control with forward primer (5′ GATTACTGCTCTGGCTCCTAGC 3′) and reverse primer (5′ GACTCATCGTACTCCTGCTTGC 3′). Each experiment was performed three times independently. The average cycle threshold of the duplicate measurements was calculated. The 2^-ΔΔCT^ method was used to quantify the relative gene expression in the L-Arg treatment group compared with the control group [13]. All quantitative PCR procedures, including primer design, validation of the PCR environment, and quantification were performed according to the Minimum Information for Publication of Quantitative Real-Time PCR Experiment guidelines [14].

### Determination of nitrite (NO_2_^−^) concentrations

Concentrations of NO_2_^−^ from the supernatants of splenocyte culture were measured using the Griess reaction [10]. Briefly, 100 μL of each supernatant was incubated with 100 μL of Griess reagent for 10 min at room temperature. NO_2_^−^ concentration was determined by measuring the optical density at 550 nm (A_550_) and comparing these values to the standard curve of NaNO_2_ solution.

### Measurement of intracellular reactive oxidant species (ROS) in spleen cells

ROS quantification was performed by measuring the oxidative conversion of 2,7-dichlorodihydrofluorescein diacetate (DCFH-DA) by peroxide. Briefly, 10^6^ splenocytes were incubated with DCFH-DA for 20 min, after which 2,7-dichlorofluorescein (DCF) fluorescence was detected by flow cytometry with an excitation wavelength of 488 nm and an emission wavelength of 530 nm. All measurements were repeated in triplicate. The resulting data were processed with CellQuest (BD Biosciences, San Diego, CA, USA).

### In vitro zygote and ookinete development assay

*P. yoelii* zygotes and ookinetes were examined using the 24 h ookinete culture assay [15]. Briefly, blood was harvested from mice at 3 days post-infection, with the D-Arg treatment group serving as the control. Ten μl of each blood sample were combined with 90 μl of ookinete culture medium and incubated for 24 h at 24 °C. After centrifugation of the culture, the pellet was resuspended in 50 μL phosphate-buffered saline (PBS), and 1 μl aliquots were spotted onto glass slides (Matsunami glass IND., LTD, Osaka, Japan), air-dried, and fixed with ice-cold acetone. Zygotes and ookinetes were detected by indirect immunofluorescence assay (IFA) with anti-Pys25 antibody. Slides were first blocked with PBS containing 5 % non-fat dry milk for 30 min at 37 °C and then incubated with a monoclonal anti-Pys25 antibody (1:200) for 60 min. After rinsing with PBS, the slides were incubated with fluorescein isothiocyanate (FITC)-conjugated goat anti-mouse IgG antibodies (Tago; Camarillo, CA) for 30 min at 37 °C. After rinsing with PBS, the slides were mounted under a cover slip in bicarbonate-buffered glycerin, and observed under a fluorescence microscope. The total numbers of zygotes and retort ookinetes formed per microlitre of the aliquots were counted.

### Mosquito rearing and infection with *P. yoelii*

*Anopheles stephensi* mosquitoes were maintained at 24 °C and 75 % humidity with a 12 h light/dark cycle. Adult mosquitoes were kept on a 5 % sucrose solution. Mosquitoes were starved overnight prior to experiments and were allowed to feed on infected mice for 30 min on day 3 post *Py*17XL infection. Engorged mosquitoes were isolated, maintained at 24 °C, fed on 5 % sucrose solution, and dissected for oocysts 9 days after the blood meal.

### Statistical analysis

Statistical significance of differences was determined using the Student’s *t*-test and Mann–Whitney *U*-test. *P*-values less than 0.05 were considered significant.

## Results

### Levels of *iNOS* and *ARG1* transcription

**Fig. 1:**
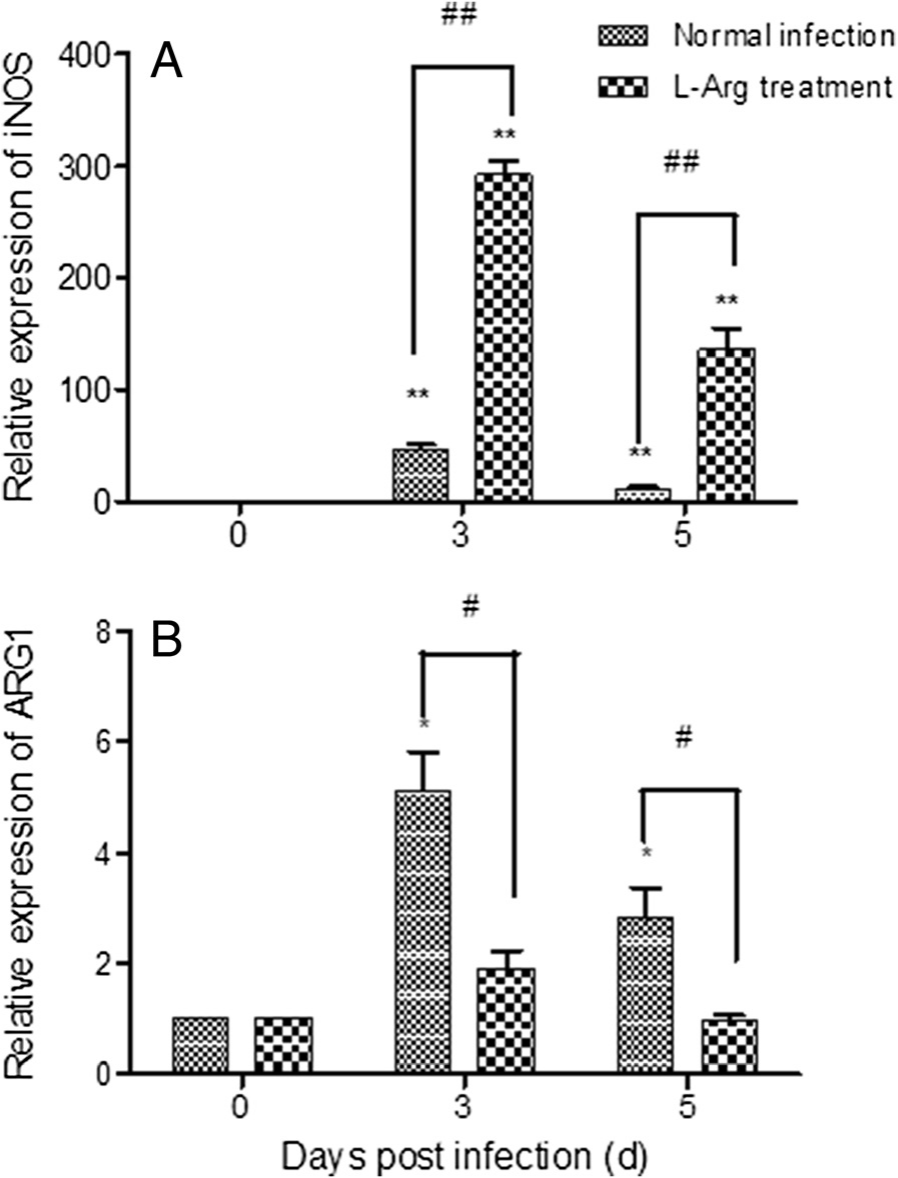
Effects of L-Arg treatment *iNOS* (**a**) and *ARG1* (**b**). The relative abundance of *iNOS* and *ARG1* in spleen cells were measured by quantitative real-time RT-PCR. Spleen cells were collected from mice prior to *Py*17XL infection (0), and 3 and 5 dpi. Results are representative of three independent experiments. For each experiment, three mice were used per group. Values represent the mean + SEM.* and ** indicate significant difference between *Py*17XL infected (3 d and 5 d) and uninfected mice (0 d) at *P <* 0.01 and *P* <0.05 (*t-*test), respectively. # and ## indicate significant difference between D-Arg pretreated control (Normal infection) and L-Arg pretreated mice (L-Arg treatment) at *P <* 0.01 and P <0.05 (*t-*test), respectively

Consistent with our previous finding [9], L-Arg supplement significantly reduced the parasitemia at 5 and 7 dpi, and significantly prolonged the survival of *Py*17XL-infected mice (data not shown). The expression of *iNOS* and *ARG1* in the spleens of uninfected and *P. yoelii*-infected mice was determined using quantitative RT-PCR (Fig. 1). Compared to uninfected mice, *Py*17XL infection resulted in a significant increase of *iNOS* transcript at 3 and 5 days post infection (dpi) (Fig. 1a, *P* < 0.01, *t-*test). L-Arg pretreatment significantly increased the *iNOS* transcript levels compared with the D-Arg pretreated group (Fig. 1a, *P* < 0.01). In parallel, compared with the uninfected group, the control *P. yoelii*-infected group also showed higher levels of the *ARG1* transcript (Fig. 1b, *P* < 0.05). However, L-Arg pretreatment led to a significant decrease of the *ARG1* transcript compared to the untreated control group (Fig. 1b, *P* < 0.05), resulting in a similar level of *ARG1* mRNA as in the uninfected group.

### Levels of NO and ROS

**Fig. 2:**
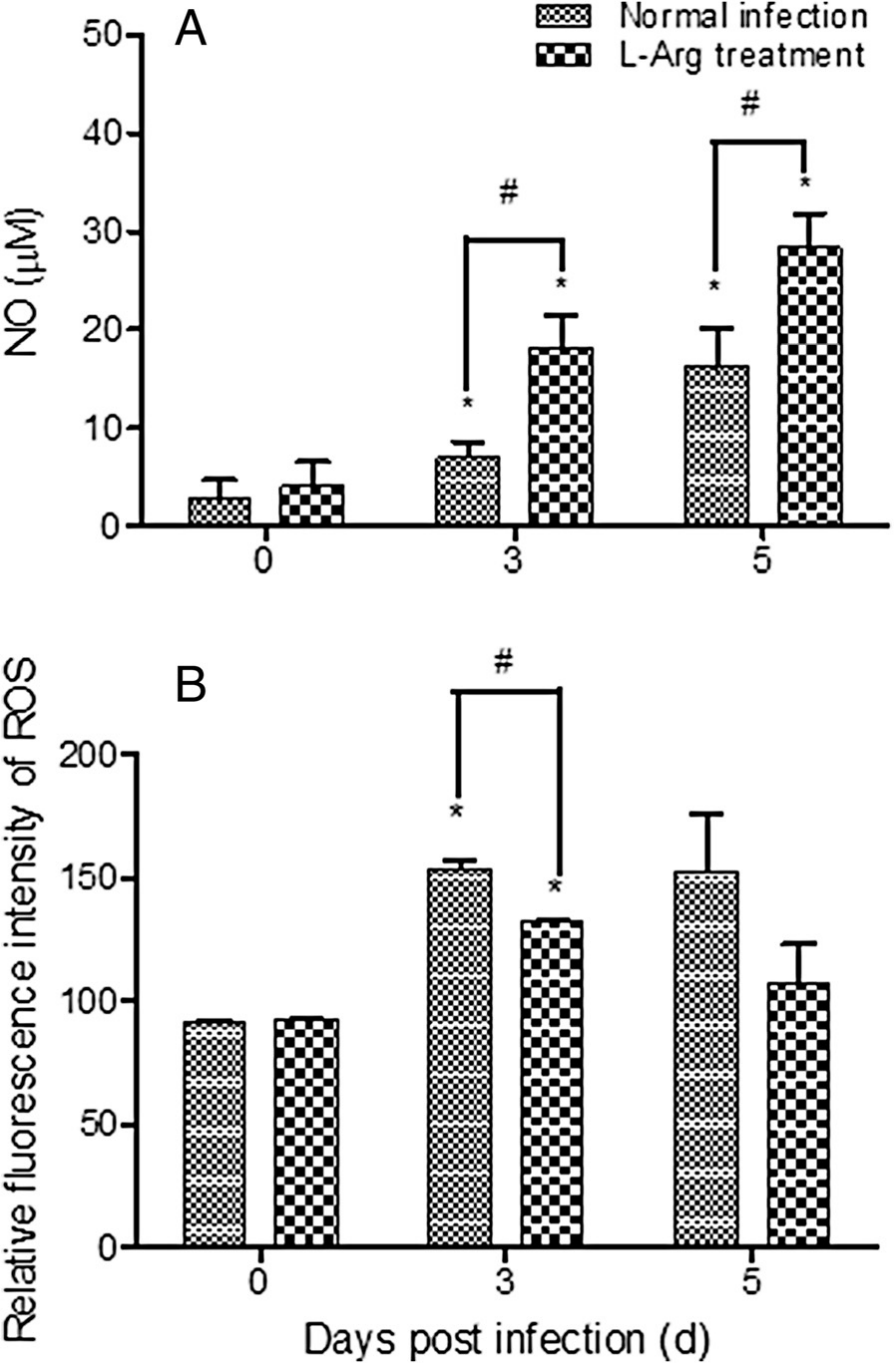
Effects of L-Arg treatment on NO (**a**) and ROS (**b**) production in spleen cells. **a** Supernatants were prepared from splenocyte cultures, and concentrations of NO_2_^−^ were detected using the Griess reaction. **b** ROS production was measured by flow cytometry. Spleen cells were collected from mice prior to *Py*17XL infection (0 d), and 3 and 5 dpi. Results are representative of three independent experiments. For each experiment, three mice were used per group. Values represent the mean + SEM. * indicates significant difference (*P <* 0.05, *t-*test) between *Py*17XL infected (3 d and 5 d) and uninfected mice (0 d). # indicates significant difference (*P* < 0.05, *t-*test) between D-Arg pretreated control (Normal infection) and L-Arg pretreated mice (L-Arg treatment)

We measured NO production in the culture supernatants of spleen cells isolated from L-Arg pretreated and control mice. The levels of NO_2_^−^ gradually increased from 3 to 5 dpi (Fig. 2a, *P* < 0.05). The L-Arg pretreatment group produced significantly higher amounts of NO than the control group (*P* < 0.05). Similarly, ROS levels also increased after *Py*17XL infection. However, ROS levels in the L-Arg group were lower than that in the control infected group, although the difference was only statistically significant at 3 dpi (Fig. 2b, *P* < 0.05).

### Effects of L-Arg on parasite transmission

**Fig. 3:**
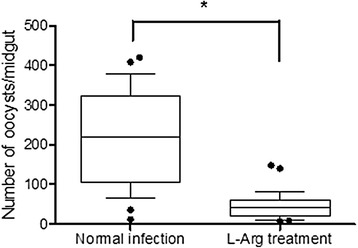
Effects of L-Arg on the infection of *A. stephensi* by *P. yoelii*. In each treatment, 20–30 mosquitoes were dissected and the number of oocysts per midgut was determined (median and interquartile range). * indicates significant difference (*P* < 0.05, Mann–Whitney *U*-test) between D-Arg pretreated control (Normal infection) and L-Arg pretreated mice (L-Arg treatment)

In addition we compared the transmission-blocking effects of L-Arg pretreatment in mice infected with *Py*17XL. At 3 dpi, infected blood was used for in vitro culture of zygotes and ookinetes and infected mice were used for mosquito feeding. The numbers of zygotes and ookinetes formed were significantly reduced in the L-Arg pretreated group compared to the control group (Table 1, *P* < 0.05). Similarly, oocyst density was reduced by 89.1 % in mosquitoes fed on L-Arg pretreated BALB/c mice compared to untreated control mice (Fig. 3).

**Table 1:** L-Arg pretreatment inhibits formation of zygotes and ookinetes

Mice	Number of zygotes (Mean ± SEM)	Number of ookinetes (Mean ± SEM)
Normal infection	65.5 ± 6.4	20.0 ± 2.8
L-Arg pretreatment	5.0 ± 4.2##	2.5 ± 2.1#

Note: # and ## indicate significant difference between D-Arg pretreated control (Normal infection) and L-Arg pretreated mice (L-Arg treatment) at *P* < 0.05 and *P* <0.01 (*t-*test), respectively

## Discussion

NO, produced endogenously through the action of NOS on its substrate L-Arg, plays a critical role in defending against infections. At low concentrations, NO acts as a signaling molecule to enhance the functions of the immune cells, while at high concentrations, NO directly inhibits or kills the pathogens. During malaria infection, supplementation of L-Arg has been shown to improve the protective immunity during early-stage *Py*17XL infection [9]. Here we show that L-Arg supplementation not only enhances the protective immunity against blood stage malaria parasites, but also has a transmission-blocking effect.

Resistance to blood-stage malaria infections depends on the ability of the host to mount an early effective Th1 immune response in order to control parasite growth [16]. This response is predominantly characterized by IFN-γ secretion and effective NO production [17]. Recruitment and activation of monocytes and macrophages are essential for this process [18, 19]. During phagocytosis, macrophages generate highly toxic reactive ROS and reactive nitrogen species (RNS), which damage intracellular components. In this heightened immune state, addition of L-Arg further enhances the Th1 response [9]. This effect is linked to the significantly increased expression of *iNOS* and elevated NO production in splenocytes. In contrast, though *ARG1* transcription was also induced during parasite infection, L-Arg pretreatment suppressed *ARG1* expression. As L-Arg pretreatment did not lead to enhanced ROS production in macrophages, the RNS pathway may play a more important role in parasite clearance.

We have shown that increased NO production inhibits asexual parasite growth in infected mice, which may be partially due to improved host immunity [9] and reduced expression of parasite invasion molecules such as the apical membrane antigen 1 (AMA1) [20]. Through the injection of an L-Arg analog, it was shown that the reduced gametocyte infectivity to mosquitoes during *P. yoelii nigeriensis* infection was due to reactive nitrogen intermediates [21]. In this study, we showed that L-Arg supplementation was detrimental to the sexual stages of the malaria parasites, resulting in decreased formation of zygotes and ookinetes in vitro and oocysts in vivo*.* This result is consistent with our earlier in vitro study of the effect of NO on gametocytes, suggesting that the L-Arg supplementation effect is likely due to enhanced NO production of the immune cells. Taken together, L-Arg supplement, as an inexpensive and safe supplement, not only promotes the protective immunity against asexual-stage malaria parasites, but also has transmission-blocking activity, which could be employed to interrupt the malaria transmission cycle. It is noteworthy that L-Arg supplement does not improve the outcome of experimental cerebral malaria in rodents [22], but significantly reverses endothelial dysfunction in human malaria [8], suggesting some differences exist in the two systems. Therefore, further investigations are needed to determine the effect of L-Arg on transmission blocking during human malaria.

## Conclusions

L-Arg pretreatment could enhance host immunity against blood-stage malaria parasites and inhibit sexual development of parasites in mosquitoes. These effects are likely due to increased transcription of *iNOS* and production of NO. These findings suggest that L-Arg as an inexpensive and safe supplement may represent a novel adjunct treatment against malarial infection.
